# Binderless Thermal Insulation Panels Made of Spruce Bark Fibres

**DOI:** 10.3390/polym13111799

**Published:** 2021-05-29

**Authors:** Jakob Gößwald, Marius-Cătălin Barbu, Alexander Petutschnigg, Eugenia Mariana Tudor

**Affiliations:** 1 Forest Products Technology and Timber Construction Department, Salzburg University of Applied Sciences, Markt 136a, 5431 Kuchl, Austria; jgoesswald.htw-m2020@fh-salzburg.ac.at (J.G.); cmbarbu@unitbv.ro (M.-C.B.); alexander.petutschnigg@fh-salzburg.ac.at (A.P.); 2 Faculty of Furniture Design and Wood Engineering, Transilvania University of Brasov, B-dul. Eroilor nr. 29, 500036 Brasov, Romania; 3 Institute of Wood Technology and Renewable Materials, University of Natural Resources and Life Sciences (BOKU), Konrad Lorenz-Straße 24, 3340 Tulln, Austria

**Keywords:** tree bark fibre, thermal insulation panels, thermal conductivity, self-bonded boards, zero formaldehyde content

## Abstract

Tree bark is a by-product of the timber industry available in large amounts, considering that approximately 10% of the volume of a tree stem is bark. Bark is used primarily for low-value applications such as heat generation or as mulch. To the best of our knowledge, this study is the first one that scrutinises thermal insulation panels made from spruce bark fibres with different densities and fibre lengths manufactured in a wet process. The insulation boards with densities between 160 and 300 kg/m^3^ were self-bonded. Internal bond, thermal conductivity, and dimensional stability (thickness swelling and water absorption), together with formaldehyde content, were analysed. The thermal properties of the boards were directly correlated with the density and reached about 0.044 W/m*K, while the internal bond was rather influenced by the fibre length and was relatively low (on average 0.07 N/mm^2^). The water absorption was high (from 55% to 380%), while the thickness swelling remained moderate (up to 23%). The results of this study have shown that widely available bark residues can be successfully utilised as an innovative raw material for efficient eco-friendly thermal insulation products.

## 1. Introduction

Bark is the outer layer of trees, divided into two anatomically different layers, the outer bark, whose primary purpose is the protection of the underlaid tissues, and the inner bark that transports the assimilation products from leaves to the root with active tissues close to cambium [1]. Various types of extractives (especially carbohydrates) are included in the tree bark [2,3].

Today, in Europe, the majority of the available bark is used for bioenergy production or is used for even less value-added purposes like composting and incinerating [4]. Tree bark can be superiorly utilised as raw material, for example, as a filler in urea formaldehyde adhesives [5,6,7,8] to replace wheat flour, reducing in this way the formaldehyde emissions. Extractives and chemical compounds of the bark offer applications as medicine, plastics, or aggregates [9]. Some tree species also allow utilisations as tissue [10,11,12].

For particleboards (PB), the use of recycled wood or other lignocellulose materials is well established [13]. Due to its availability, bark, which encompasses about 10% of the stem volume [14], has interesting potential in bark-based composites. Muszynski and McNatt [15] showed that with up to 30% spruce and pine bark, it is possible to produce particleboards with acceptable properties. Blanchet et al., 2000 [15] indicated that even larger wood particle proportions could be replaced, especially when using the inner bark of birch trees [16]. With treatments in hot water, the mechanical properties of particleboards containing tree bark can be modified [17].

Because bark is a fibrous tissue, the production and utilisation of black spruce bark fibres in the core layer of medium density fibreboards (MDF) were evaluated by Xing et al. [18,19]. According to this study, it can be stated that black spruce bark can be an auxiliary material for the core layers of MDF.

Bark fibres can be produced by thermo-mechanical refining [19,20], while bark particles can be obtained with various methods, for example, shredding or hammer-milling [15,21].

One purpose of bark is the protection of the tree from external influences like moisture loss and temperature changes; therefore, a natural optimisation of bark toward these insulation properties has already been the subject of study of many research teams [22]. Bark composites with clay as a binder open a new means of the manufacture of panels with enhanced resistance to fire [23]

The thermal conductivity (TC) of bark-based panels was evaluated in various studies, for example, in insulation boards bonded with natural tannins [21,24,25] or with larch, pine, spruce, fir, and oak tree bark resinated with urea-formaldehyde, melamine urea-, and tannin-based adhesives. The evaluation of the effects of particle orientation in insulation panels for larch bark showed lambda (λ) values (TC) between 0.056 to 0.1 W/m*K [25].

Regarding the differences between inner and outer bark with regard to thermal properties, some studies indicate that the inner bark is able to insulate better compared to the outer bark, especially in trees that contain large fibres [12]. Another important issue for the implementation of tree bark in added-value applications is its comminution type [26].

With reinforced surfaces, the mechanical properties of bark insulation boards can be enhanced [27]. Lower TC (0.045 W/m*K) was achieved after alkaline extraction of poplar bark [14]. Apart from thermal insulation, bark-based composites can be used as sound-absorbing panels. At densities below 500 kg/m^3^, the bark composites had better sound absorption than most other wood-based products [28,29].

Another advantage of bark might be its suitability for self-bonded boards. The studies of [15] and [30] that dealt with the manufacture of self-agglomerated PB based on bark observed the effect of particle plasticisation and extractive polymerisation on bark particles’ self-bonding.

Burrows (1960) [31] studied the properties of self-bonded Douglas-fir PB and suggested that the plasticisation mechanism may occur due to the lignocellulosic character of bark and due to the presence of water as a plasticiser. This premise is complemented by the conclusion of [32] regarding the lignocellulosic materials with lightweight molecules (lignin polymers, non-crystalline cellulose, and hemicellulose) that permit softening at a convenient temperature for producing a plasticised matrix that can connect particles in self-bonding panels.

In the present study, the suitability of spruce bark fibres for use in low-density insulation panels was analysed. It was assumed that the reduction in density would decrease the TC of the boards. Due to extractives and the fibrous nature of the bark material, stable boards can be produced in a wet process, without supplementary resins. Subsequently, the influence of the fibre length and density was examined, together with formaldehyde content.

## 2. Materials and Methods

The bark was sourced from fresh spruce trees (*Picea abies*) from a sawmill in Altötting (Germany) with a diameter over 20 cm. The logs were debarked using high-pressure water jets provided by a Kärcher HD 1090 (Winnenden, Germany). Due to the high water pressure (200 bar), the breakdown of bark resulted in larger pieces and fibrous material, as observed by Krivo et al. (1983) [33]. Since spruce bark in contact with air dries and oxidates quickly, visible due to a change in colour from white to brown within some hours, air contact was avoided where possible.

**Figure 1:**
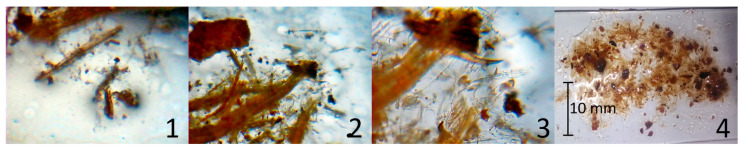
Bark fibres included in the length class 1.6 mm; magnification: 4×, 4×, 10×, reference picture; dependencies: **1** and **2** are details of **4**; **3** is a detail of **2**.

The wet fibre material was fractionated using sieves of 7, 4, and 1.6 mm. The wet sieving provides the advantage that the fibres can be easily processed, because the fibres clump together during drying. Three types of fibre bundle lengths were chosen for this experimental design, 1.6, 4, and 7 mm, which correspond to the mesh size of the sieves. Due to anatomical differences between phloem and phellem, the brittle parts of the bark degraded into small particles, while the fibrous parts of the bark formed fibre bundles and single fibres. An example of the composition of 1.6 mm fibres is presented in Figure 1.

The wet process was applied for the low-density bark fibre boards without using additives or adhesive. At the laboratory scale, the fibres were mixed with 8 L water and were subsequently dehydrated on a fabric supported by a sieve and further squeezed using an overlaying sieve with 9 kPa. The wet board with the size of 30 cm × 30 cm was dried in a Binder (Tuttlingen, Germany) oven at 103 °C for 24 h. Table 1 shows the density and fibre length class of the bark fibre insulation boards. The panel type is coded as follows: the letter A is for bark fibre length 1.6 mm, letter B for 4 mm fibre length, and C for 7 mm fibre length. To the codification belongs also the target density (200 and 250 kg/m^3^).

Due to the shrinking during drying, the boards need to be calibrated (milled to obtain a homogeneous and constant thickness) and cut to size to measure the thermal conductivity, carried out using the single-plate λ-Meter EP 500e of the Lambda Messtechnik GmbH (Dresden, Germany) according to EN 12677:2001 [34]. After testing, 50 mm × 50 mm samples were used to determine the internal bond according to EN 1607:2013 [35] with a Zwick Roel Z250 universal testing machine (Ulm, Germany). The dimensional stability (thickness swelling/water absorption after 24 h) was determined according to EN 317:2005 [36], as well as the free formaldehyde content with the perforator method EN ISO 12460-5:2015 [37]. All boards were cut in compliance with EN 326: 1994 [38] and conditioned at 20 °C and 65% relative air humidity for one week, until constant mass was reached, before the testing. The results were analysed using Python software. A regression analysis with all variables at 5% significance was performed in combination with an ANOVA and a test of heteroskedasticity.

## 3. Results and Discussion

Before the first samples were tested, the easy processability of bark fibre boards was observed, due to the efficient grindability and the cuttability with the cutter knife, especially at fibre bundles length of 1.6 mm.

### 3.1. Formaldehyde Content

The formaldehyde content of the boards with fibre bundle length of 4 mm and 180 kg/m^3^ density was determined, according to EN ISO 12460:5:2015 at the company Kaindl (Wals, Austria), to be 0 mg/100 g. Since bark has the ability to bind formaldehyde [6], values significantly under 1 mg were expected; however, similar studies based on larch bark panels showed slightly higher formaldehyde contents [6,39]. With no formaldehyde content, these boards are included in the super E0 classification (<1.5 mg/100 g). The zero value for the formaldehyde content may be attributed to the high amount of lignin in the chemical composition of tree bark [40]. The lignin content of spruce bark ranges from 26% [41] to 37% [42]. Due to their phenolic nature, bark tannins can react with formaldehyde as a substitute for phenol in the formation of wood adhesives, which can be confirmed by the low formaldehyde content [43].

### 3.2. Physical Properties

The results of the physical and mechanical properties of the insulation panels made of spruce bark fibres are shown in Table 2.

### 3.3. Thickness Swelling and Water Absorption after 24 h

**Figure 2:**
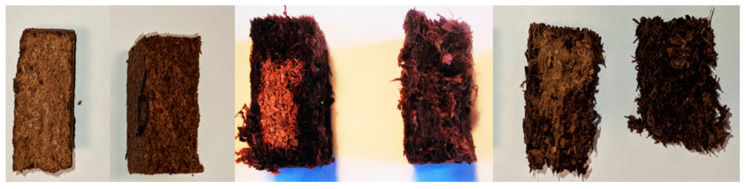
Water penetration over the cross section of the tested sample: fibre length 1.6 mm, wet and dry (**left**); fibre length 4 mm, wet and dry (**middle**); and fibre length 7 mm, wet and dry (**right**).

For the analysis of the thickness swelling (TS) and water absorption (WA) after 24 h measured according to EN 317:1993, two cases need to be considered. In some samples for all fibre bundles lengths (1.6, 4, and 7 mm), dry spots could be located, which show a different behaviour compared to the wet samples (Figure 2). Dry spots seem to occur at the lowest densities of each fibre bundle length. Such an effect could not be observed by similar studies, which rather show a linear corelation [21]. To the best of our knowledge, this study is the first attempt to investigate the low-density bark fibre boards, so the incidence of dry spots is specific to this material. Additionally, the values for 7 mm fibre bundles’ length need to be considered less precise due to the very low values of internal bond (Subchapter 3.4) that influenced the measuring error of TS and WA.

**Figure 3:**
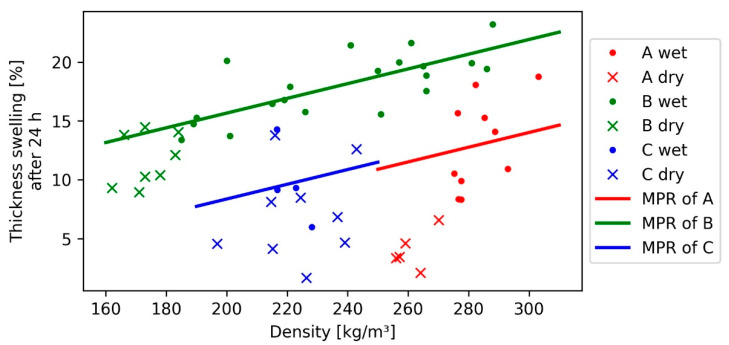
Multiple polynomial regression (MPR) of thickness swelling after 24 h for each bark fibre length class with the density; only the regression for wet samples is depicted.

For TS and WA, after 24 h, a multiple polynomial regression (MPR) analysis was performed with the significant variables: intercept, density, fibre length, and fibre length squared. The regression of TS for the wet samples was highly significant and positively correlated, while R² showed a value of 0.61 (Figure 3). With a polynomial regression of the fibre length, it was possible to model the thickness swelling for all fibre bundle classes. A typical TS for wood-based panels shows a positive correlation with the fibre thickness [13]. Since with longer fibres an increased fibre thickness can be expected, when using the described defibration method, the 7 mm fibre bundle length (C) did not follow this prediction. Due to the reduced IB and density of this panel, combined with the low slenderness ratio, large holes and less felted regions can be expected, compared with 1.6 (A) and 4 mm (B) fibre lengths. This characteristic can affect the TS, since holes are a favourable field for the swelling fibres. For the dry samples, the regression was still significant; however, R^2^ was 0.47, most likely due to grouped density range of the dry samples. Additionally, the dry samples of fibre length 1.6 (A) and 4 mm (B) seem to have the lowest density of their classes. The difference in TS between wet and dry samples was not as strong as in the WA. It is assumed that the core fibres soaked up just enough water to stay under the equivalent of the fibre saturation point in wood. The highest TS 24 h value of 23% could be found for 4 mm fibre length (B), whereas the lowest values with 3.3% and 1.6% could be found for 1.6 mm (A) and 7 mm (C) fibres. Medium-sized fibres seem to have a disadvantage in terms of TS. The smaller density variation for 1.6 mm (A) and 7 mm (C) fibre length makes it hard to predict whether the TS and WA trends foreseen by the model are stable at other density levels, too.

**Figure 4:**
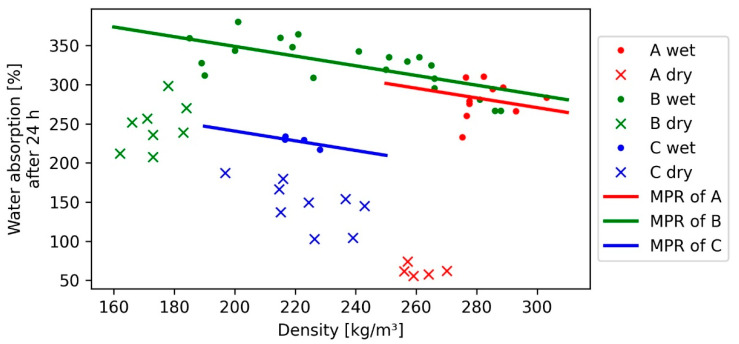
Polynomial regression (MPR) of water absorption after 24 h for each bark fibre length class (FLC) with the density; only the regression for wet samples is depicted.

The water absorption (WA) after 24 h for the wet sample values varied from 217% to 380%, according to the production method and the absence of adhesive or hydrophobic additives. In contrast, in dry samples with 1.6 mm fibre length, values under 55% were achieved. Figure 4 shows relatively similar WA of wet samples of 1.6 mm (A) and 4 mm (B). As explained in previous subchapter TS, the WA of the C samples (7 mm) might be underestimated. If the WA of 7 mm fibre bundles is excepted from the measurement series, it could be stated that the fibre length seems to have little effect on the water absorption. Between the dry and wet samples of each fibre length, a sudden drop can be identified, which increases with decreased fibre length, indicating a polynomial corelation to the density, caused by the dry samples. In that case, it can be expected that with smaller fibre length and lower densities, the water absorption can be decreased, which in most cases is a favourable material property. The decreased water absorption of dry samples is considered to be caused by the increased pore size of lower density samples, which come along with a reduced capillary force, so the water could not be soaked into the sample. Assuming that, compared to wood, bark contains more hydrophobic substances, such as suberin [14], an improvement of water repellence can be observed in such composites. The regression of the WA shows a negative correlation with the density, which is also more significant and shows less prediction error due to an R^2^ of 0.73. Since similar studies [25] also show a negative correlation, the measured data correspond to the expectations. For the dry samples, a highly significant model with an R^2^ of 0.88 was obtained. As Figure 4 depicts, the dry samples appear grouped and do not show overlapping density areas like the wet samples. The accuracy of the model regarding the negative correlation of the dry samples model needs to be questioned. On the one hand, fibre bundles of 7 mm (C) and (A) indicate the negative correlation. On the other hand, the fibre bundles of 4 mm (B) seemed to follow more of a polynomial behaviour towards the density and therefore a positive correlation with the density in the corresponding interval.

### 3.4. Internal Bond

**Figure 5:**
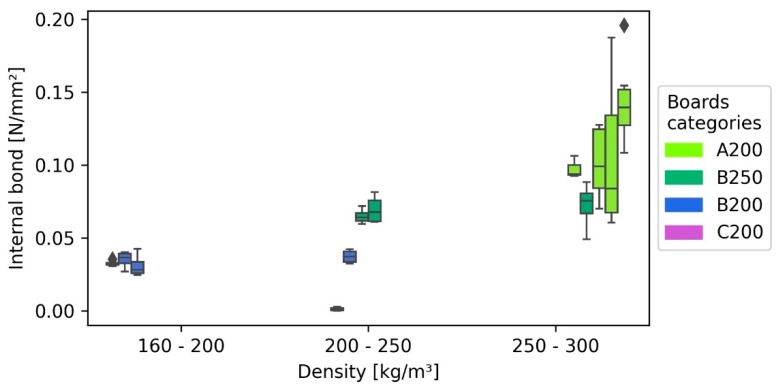
Internal bond of the insulation panels with 1.6, 4, and 7 mm bark fibre length as a function of density.

The internal bond ranged between 0.2 and 0.0 N/mm^2^; however, only boards with 1.6 mm fibre bundle length (A) were able to achieve values over 0.1 N/mm^2^ (Figure 5). One reason is the higher density of those boards (277 kg/m^3^ average), caused by the proportionately higher shrinking of the board during the drying process.

**Figure 6:**
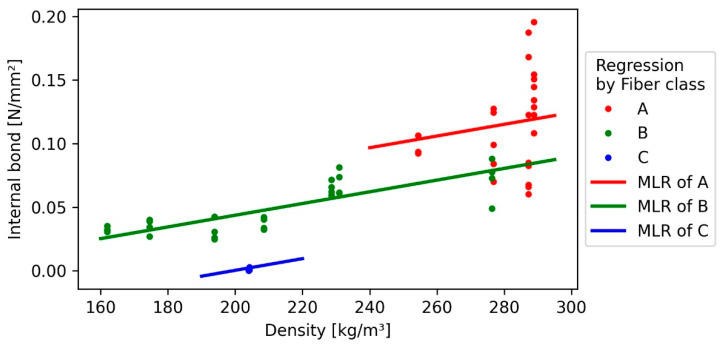
Multiple linear regression (MLR) of internal bond towards the independent variables density and bark fibre length (lines) and measured values (points).

As depicted in Figure 3, the internal bond of C200 boards (7 mm fibre bundle length) shown in comparison to the other boards’ values close to 0.0 N/mm^2^. This behaviour can be explained due to thicker fibres that decreased the homogeneity and, as a consequence, the self-bonding capacity [13]. Within the other board categories (B200, B250, and A200), the IB showed almost no correlation with the density. Because such a correlation is typically for wood-based panels [13], this indicates together with the asymmetry of some boxplots (due to the scattering of internal bond, especially for the fibre length 1.6 mm (A)), that this effect is caused by variation within the panels. Because this variation increases with the density, heteroscedastic effects were detected by the “White-test” for heteroscedasticity within the regression model. Because a constant amount of process water was used during the panel manufacturing, the solid content of the fibre-water suspension of boards with higher density was subsequently higher than those of panels with lower density, therefore inducing more variation to the higher density boards. Simple linear regressions (SLR) involving only one fibre class do not show heteroscedasticity, and it is therefore a result of the larger dataset of the multiple linear regression (MLR). However, the variation of fibre length 1.6 mm seems to be higher than that of fibre length of 4 mm (Figure 6), slightly indicating that smaller fibres might be more prone to irregular fibre distribution during the forming process than longer fibres at the same density.

The MLR shows an R^2^ of 0.7, while both variables (fibre length and density) are highly significant. Due to the uneven distribution of the samples of fibre length of 1.6 mm (A) and 7 mm (C), the results of the regression are only valid in areas, where data points are available. Additionally, its explanatory power is decreased by its heteroscedasticity. Irregular fibre distribution can lead to a decrease in the average performance of the board and an increased variation within the board [44]. Therefore, heteroskedasticity in the model indicates an underestimation of the slope of the model.

### 3.5. Thermal Conductivity (TC)

**Figure 7:**
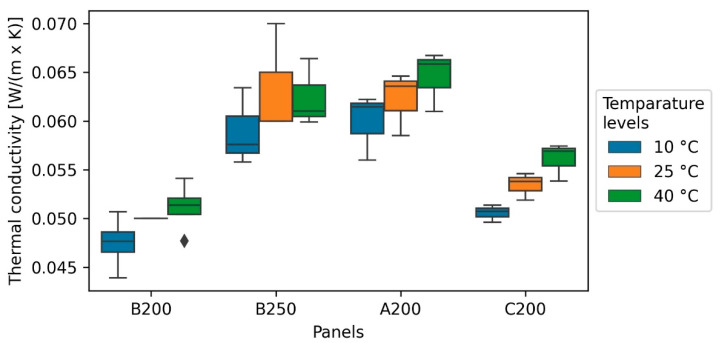
Thermal conductivity of bark fibreboards under different temperature levels (10, 25, and 40 °C).

The lowest TC was measured with 0.044 W/(m*K) and a density of 162 kg/m^3^ at the fibre length of 4 mm (B), whereas the highest value of 0.063 W/(m*K) was measured in the same fibre length at 276 kg/m^3^. In general, boards with a lower variation in density also showed a lower TC and a larger asymmetry in the boxplots (Figure 7), as also the coefficient of variation indicates, since both vary in similar intervals of 2–7% for TC and 2–11% for the density. Bark fibre boards performed in terms of TC around 8% better at lower temperature (10 °C) than at higher temperature (40 °C), as can be seen in Figure 7.

**Figure 8:**
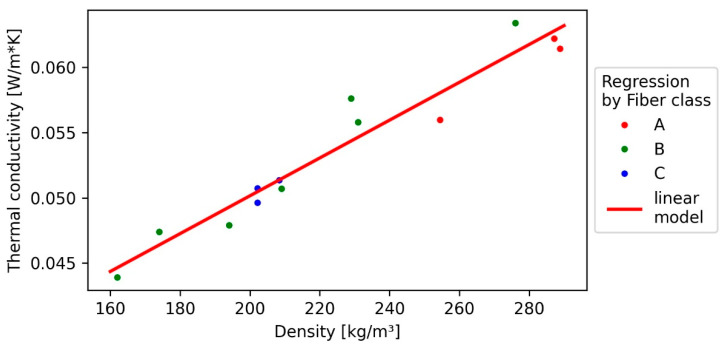
Simple linear regression (SLR) of thermal conductivity towards density (line) and measured values (points).

Similar to the IB, the TC also correlates highly significantly with the density, but in contrast to it, the fibre length was not significant, resulting in a simple linear model. However, some studies indicate a slight influence of the fibre length [45]. To exclude any influence of the fibre length, a larger data set is required. However, as Figure 8 presents, the regression model fits the data with R² of 0.94 in comparison to the other models quite well, most likely because density variation within the boards does not influence the outcome strongly. For the same reason, the model is not affected by heteroscedasticity like the IB model, even if it is possible for irregularities in the panel forming to also influence the TC to some extent.

Compared to similar studies, the slope of the model (0.013 W/m*K/100 kg/m^3^) is 54% higher (Figure 8) [21]. The TC of the spruce bark fibre insulation panels is at least 15% higher compared to mineral wool and polystyrene (approximately 0.03 W/m*K) but seemed to have a benefit over particle-based insulations, as reported by Kain et al., 2020 [21].

## 4. Conclusions

To the best of our knowledge, this research is the first attempt to investigate low-density insulation boards made of bark fibres.

The results of this study showed that the thermal insulation properties of bark fibre insulation boards can reach thermal conductivity from 0.044 W/m*K (at a density of 164 kg/m^3^) to 0.063 W/m*K (276 kg/m^3^), being significantly influenced by the density. These TC values are comparable to established insulation boards based on cork or wood fibres [46]. The effect of the fibre length was not significant for the TC, as observed in previous studies [45]. However, with spruce bark fibres, it is possible to achieve lower thermal conductivity than with particle-based bark panels [21].

The internal bond was furthermore influenced significantly by the length of the bark fibres bundles. However, the used model has a large variation and is therefore less reliable. The variances are most likely caused by an insufficiently low solid content during the board production, but the wet process has still proved its ability to produce bark fibre-based insulation boards without adding resins, therefore indicating a sufficient self-agglomeration and sticking of bark fibres.

However, without additional hydrophobic additives, the water absorption after 24 h can rise up to 380%, while the thickness swelling after 24 h remains under 25%. At lower density, bark fibre boards did not show a complete wetting anymore, which goes along with a drop in water absorption down to 55% and a reduced thickness swelling.

Based on the measured thermal conductivity and zero formaldehyde content, bark fibre insulation panels might be able to compete with conventional insulations if the density can be further reduced, but also applications regarding its acoustic insulation are thinkable [28]. To answer these questions, further research is necessary regarding fields such as the impacts of bark species and bark quality as well as other production methods or properties crucial to certain applications such as its protection capability towards structure-borne noise or fire.

## Figures and Tables

**Table 1:** Experimental design of bark fibre boards with three fibre bundle lengths (1.6, 4, and 7 mm) and two density levels (200 and 250 kg/m^3^).

Insulation Panel (Target Density)	Density (kg/m^3^)	Fibre Length (mm)	Boards Number
A200	277	1.6	3
B250	245	4	3
B200	185	4	4
C200	204	7	3

**Table 2:** Physical and mechanical properties of the spruce bark fibre insulation boards (values with the same letter (a, b, c, d) are not significantly different ANOVA, post-hoc Tukey HSD, *p* = 0.05; standard deviation in parentheses).

Sample	TS %	WA %	TC 10 °C mW/(m*K)	TC 25 °C mW/(m*K)	TC 40 °C mW/(m*K)	IB N/mm²	Density kg/m^3^
A200	10.0 ^b^ (5.4)	207 ^b^ (108)	59.9 ^c^ (3.4)	62.2 ^c^ (3.3)	64.5 ^c^ (3.1)	0.129 ^c^ (0.035)	277 ^d^ (19)
B250	18.6 ^d^ (3.8)	301 ^c^ (32)	58.9 ^c^ (3.9)	60.8 ^c^ (3.7)	62.4 ^c^ (3.5)	0.069 ^b^ (0.011)	245 ^c^ (27)
B200	14.4 ^c^ (3.0)	305 ^c^ (57)	47.5 ^a^ (2.8)	49.3 ^a^ (2.7)	51.1 ^a^ (2.6)	0.034 ^b^ (0.006)	185 ^a^ (21)
C200	8.0 ^a^ (3.8)	172 ^a^ (45)	50.6 ^b^ (0.9)	53.4 ^b^ (1.4)	56.1 ^b^ (1.9)	0.009 ^a^ (0.013)	204 ^b^ (4)

## Data Availability

It is not the case, no datasets.
